# Data-driven subtyping and staging of ALS: A multicenter, longitudinal, deformation-based morphometry study

**DOI:** 10.1162/IMAG.a.1264

**Published:** 2026-06-04

**Authors:** Isabelle Lajoie, Sanjay Kalra, Mahsa Dadar

**Affiliations:** Douglas Mental Health University Health Centre, Montreal, Quebec, Canada; Department of Psychiatry, McGill University, Montreal, Quebec, Canada; Neuroscience and Mental Health Institute, University of Alberta, Edmonton, Alberta, Canada; Division of Neurology, Department of Medicine, University of Alberta, Edmonton, Alberta, Canada

**Keywords:** subtype and stage inference, progression modeling, machine learning, longitudinal

## Abstract

Amyotrophic lateral sclerosis (ALS) is clinically and biologically heterogeneous, yet data-driven imaging subtyping approaches have rarely been validated longitudinally or linked to clinical and survival outcomes. We aimed to identify and validate distinct ALS subtypes and disease stages using deformation-based morphometry (DBM) and the Subtype and Stage Inference (SuStaIn) model, and to characterize their cross-sectional and longitudinal imaging, clinical, cognitive, and survival profiles. Data from 198 ALS patients and 144 healthy controls in the Canadian ALS Neuroimaging Consortium (CALSNIC) multicenter cohort were analyzed. Baseline regional DBM w-scores from 14 ALS-relevant regions served as input to SuStaIn to infer subtypes and stages. Longitudinal consistency of subtype and stage assignments (e.g. adherence to the expected disease evolution) was assessed using follow-up visits. Imaging and clinical trajectories were compared across subtypes using linear mixed-effects models incorporating stage and elapsed time. Associations between longitudinal variables and SuStaIn stage were estimated using mixed models, while baseline clinical and cognitive differences were assessed with ordinary least squares regression. Survival differences were evaluated using Kaplan–Meier curves and log-rank tests. SuStaIn identified one normal-appearing group (S0) and three ALS atrophy subtypes. S0 showed no baseline atrophy but exhibited longitudinal motor decline and the most favorable survival (log-rank *p* < 0.05 to *p* < 0.01). S1 exhibited classical motor/corticospinal tract-dominant degeneration, greater lower motor neuron burden, and intermediate survival. S2 showed limbic-onset atrophy progressing toward motor pathways, with preserved cognition and a milder course. S3 demonstrated extensive fronto-parietal and striatal atrophy, longitudinal motor–thalamic degeneration, and the shortest survival. Subtype and stage assignments demonstrated high longitudinal consistency (>90%). SuStaIn stage was strongly associated with widespread brain atrophy (and ventricular expansion), with the strongest effects in limbic–subcortical regions. Stage also correlated with ALS Functional Rating Scale-Revised (ALSFRS-R) decline and forced vital capacity (FVC) reduction, indicating that stage reflects disease-linked progression. This study establishes a robust, longitudinally validated model of ALS heterogeneity, showing that SuStaIn-derived subtypes define distinct disease trajectories, whereas the normal-appearing group reflects an early, structurally preserved state with a more favorable survival profile. By integrating probabilistic staging with longitudinal modeling, these findings clarify dynamic subtype-specific progression patterns and support the use of SuStaIn for biologically informed patient stratification, prognostication, and clinical trial enrichment in ALS.

## 1 Introduction

Amyotrophic lateral sclerosis (ALS) is a fatal neurodegenerative disease characterized by progressive degeneration of both upper and lower motor neurons, leading to muscle weakness, respiratory failure, and death typically within 2–5 years of symptom onset (Hardiman et al., 2017; Rowland & Shneider, 2001). Clinical presentations are highly heterogeneous regarding site of onset (which may be bulbar, limb, or respiratory), progression rate, survival time, and cognitive/behavioral involvement (Chiò et al., 2013; van Es et al., 2017). Thus, ALS lies within a spectrum (ALS-frontotemporal spectrum disorder [ALS-FTSD]); up to half of patients develop cognitive or behavioral impairment, and approximately 15% meet diagnostic criteria for the frontotemporal dementia (Phukan et al., 2012; Strong et al., 2017).

This heterogeneity complicates diagnosis, prognosis, and the interpretation of neuroimaging findings, as well as the design of therapeutic trials. Understanding subtype-specific trajectories allows for more precise intervention timing: rapid cognitive–motor decline may require early neuropsychological monitoring and multidisciplinary management, whereas “neuroprotected” individuals with slower progression could inform resilience mechanisms and targeted therapies.

Several clinical staging systems have been proposed to quantify ALS progression, most notably the King’s and Milano–Torino (MiToS) frameworks (Chiò et al., 2015; Roche et al., 2012). These systems measure how a person’s ability to function declines over time, but they mainly focus on obvious motor symptoms and do not fully reflect the underlying biological changes in the disease. In contrast, post-mortem and neuroimaging studies suggest that ALS pathology spreads along distinct neuroanatomical pathways, starting primarily in motor regions such as the precentral gyrus and corticospinal tract (CST), and progressing to extra-motor and limbic structures including the hippocampus and amygdala (Brettschneider et al., 2013; Dadar et al., 2020; Kassubek et al., 2014; Verstraete et al., 2010). Such complexity cannot be captured by linear clinical staging alone and highlights the need for data-driven, biomarker-based models that integrate both spatial and temporal dimensions of disease progression.

Magnetic resonance imaging (MRI) offers a sensitive in vivo measure of ALS-related neurodegeneration. Deformation-based morphometry (DBM), in particular, provides a voxel-wise, whole-brain measurement of regional tissue deformation, quantifying both cortical and subcortical contraction (reflecting parenchymal atrophy) and expansion (such as ventricular enlargement) (Ashburner et al., 1998). While cortical thickness may offer higher sensitivity to subtle surface changes, our recent work in frontotemporal dementia indicates that FreeSurfer-derived cortical thickness often suffers from substantially higher processing failure rates than DBM (Metz et al., 2025). Previous DBM-based studies have detected progressive cerebral atrophy and expansion in various neurodegenerative disorders (Dadar et al., 2020; Friese et al., 2010; Lajoie et al., 2025; Metz et al., 2024; Pieperhoff et al., 2022). Moreover, utilizing the Canadian ALS Neuroimaging Consortium (CALSNIC) framework, a large-scale, prospectively acquired multicenter longitudinal cohort, we previously applied DBM to investigate regional brain atrophy’s contribution to individualized survival prediction (Lajoie et al., 2025). However, these frameworks generally assume a common sequence of atrophy across the cohort, which may obscure distinct biological subtypes. Recent advances in machine learning have enabled the development of probabilistic disease progression models capable of revealing hidden subtypes and their temporal evolution within a population (Iturria-Medina et al., 2020; Young et al., 2018, 2024).

The Subtype and Stage Inference (SuStaIn) model is one such framework. SuStaIn jointly infers distinct disease subtypes, each representing a characteristic sequence of biomarker abnormalities, and assigns every participant a probabilistic stage along that trajectory (Young et al., 2018). Originally developed for Alzheimer’s disease and frontotemporal dementia (FTD) (Young et al., 2018; Vogel et al., 2021; Young et al., 2021), SuStaIn has since been applied to multiple sclerosis (Eshaghi et al., 2021), and more recently to TDP-43 pathology and ALS-FTD (Shen et al., 2023; Young et al., 2023). Yet, prior ALS applications were limited by primarily cross-sectional data, and limited validation against longitudinal clinical or imaging outcomes. Moreover, the relationship between SuStaIn-derived disease stage and both clinical and imaging measures remains largely unexplored. Notably, no prior studies have leveraged DBM within a multicenter, longitudinal framework to capture subtype-specific trajectories and evaluate the temporal consistency of SuStaIn-derived stages.

The present study addresses these limitations—and differs from our prior DBM work on survival prediction (Lajoie et al., 2025)—by characterizing disease heterogeneity and progression dynamics in ALS, applying SuStaIn to DBM-derived regional brain volumes from a large, prospectively acquired multicenter longitudinal ALS cohort (CALSNIC-1 and CALSNIC-2). Our goals were to [i] identify data-driven ALS subtypes and their progression trajectories; [ii] evaluate the longitudinal consistency of subtype and stage assignments; [iii] compare cross-sectional and longitudinal imaging and clinical features between subtypes, including survival; and [iv] examine the association between inferred disease stage and both clinical and imaging measures, accounting for elapsed time. By integrating SuStaIn with longitudinal mixed-effects modeling, this work aims to define biologically meaningful trajectories of ALS progression that integrate imaging-based staging with clinical outcomes.

## 2 Methods

### 2.1 Participants

Longitudinal MRI and clinical data were obtained from the Canadian ALS Neuroimaging Consortium (CALSNIC) multicenter cohort (Kalra et al., 2020). This dataset comprised assessments from seven research sites across Canada and the United States, collected between 2014 and April 2021, and included up to three study visits (baseline, ~4-month, and ~8-month follow-ups). CALSNIC adheres to the principles of the Declaration of Helsinki and received approval from the Health Research Ethics Boards of all participating sites. Written informed consent was obtained from all participants and the University of Alberta Health Research Ethics Board (HREB) granted approval for the protocol presented in this study. Clinical and MRI protocols were harmonized across research centers and vendors. Patients were included if they had signs of both upper motor neuron (UMN) and lower motor neuron (LMN) involvement upon initial diagnosis and met criteria for possible, probable, laboratory-supported probable, or definite ALS according to the Revised El Escorial Criteria (Brooks et al., 2000). Participants meeting clinical diagnostic criteria for ALS-FTD were excluded. Our cohort, therefore, represents the ALS-FTD spectrum (ALS-FTDS), including individuals with mild cognitive (ALSci) or behavioral (ALSbi) impairment, while excluding those with comorbid dementia. Following the exclusion criteria (detailed in the Supplementary Materials, Section A), the final study population included 198 ALS patients (198_baseline_/120_visit2_/80_visit3_) and 144 healthy controls (HCs) (144_baseline_/110_visit2_/84_visit3_). These numbers reflect methodological adjustments from our previous analysis (Lajoie et al., 2025), specifically the inclusion of patients with longer symptom durations to capture a broader progression spectrum and the application of stricter cognitive screening for healthy controls.

### 2.2 Clinical and demographic data

Clinical evaluation included the ALS Functional Rating Scale-Revised (ALSFRS-R) (Cedarbaum et al., 1999), disease progression rate (DPR), finger and foot tapping rates, Edinburgh Cognitive and Behavioural ALS Screen (ECAS) (Abrahams et al., 2014), UMN (Kalra et al., 2020) and LMN involvement, site of (first symptom) onset, forced vital capacity (FVC), symptom duration at the first MRI visit, years of education, age, and sex. Cognitive impairment was evaluated using the ECAS according to thresholds established by McMillan et al. (2022), with performance expressed as the percentage of abnormal values. This allowed quantification of cognitive deficits across multiple domains, including executive function, language, and verbal fluency (processes associated with frontal network dysfunction) as well as memory and visuospatial abilities. This approach enabled assessment of cognitive involvement across the ALS-FTD spectrum even in the absence of participants with a formal dementia diagnosis. For further details on the clinical evaluations, see Supplementary Materials, Section B.

### 2.3 Image preprocessing

All T1-weighted MRIs were processed using PELICAN (Dadar et al., 2025), our open source and extensively validated pipeline based on the open access MINC (Medical Imaging NetCDF: https://github.com/BIC-MNI/minc-tools) and ANTs tools (Advanced Normalization Tools: http://stnava.github.io/ANTs/). Pre-processing steps included denoising (Coupe et al., 2008) and intensity inhomogeneity correction (Sled et al., 1998). To ensure signal consistency across the multicenter sites, image intensities were further normalized to a 0–100 range using the MINC “volume_pol” tool, which performs piecewise polynomial histogram matching to align input images with a standard reference histogram. The images were linearly (Dadar et al., 2018) and nonlinearly (Avants et al., 2008) registered to the MNI-ICBM152-2009c template (Fonov et al., 2009). All steps were visually assessed to exclude cases with significant artifacts or inaccurate registrations. DBM maps were calculated as the Jacobian determinant of the non-linear deformation fields. DBM values greater than 1 indicate localized expansion compared with the corresponding voxels in the template, whereas values smaller than 1 indicate relative shrinkage, that is, atrophy. To minimize partial volume effects from the sulci, DBM maps were masked before calculating mean deformation across 62 regions of interest (ROIs). These ROIs comprised gray matter (GM) and ventricle regions defined by the CerebrA atlas (Manera et al., 2020), as well as white matter (WM) tracts and the corpus callosum delineated by the JHU (Wakana et al., 2007) and Allen (Hawrylycz et al., 2012) atlases, respectively.

To focus on disease-specific atrophy in ALS patients, we calculated regional w-scores at each patient visit (Farahani et al., 2025). The w-score is the adjusted difference between a patient’s observed DBM value and its predicted value normalized for age, sex, and imaging site. This predicted value is derived from a linear mixed-effects model based on data from HCs. A negative w-score indicates greater atrophy than expected levels in the HCs.

### 2.4 Subtype and stage inference modeling

The Subtype and Stage Inference (SuStaIn) algorithm is an unsupervised machine-learning framework that jointly models disease subtypes and within-subtype progression stages from cross-sectional data (Young et al., 2018). SuStaIn identifies subgroups of individuals sharing similar patterns of biomarker abnormality and reconstructs a probabilistic sequence of disease progression within each subtype. Each participant is then assigned both a most likely subtype and an estimated disease stage.

SuStaIn models disease progression as a sequence of biomarkers transitioning from normal to abnormal states, relative to a control group. In this study, biomarkers corresponded to regional brain volumes derived from DBM-based w-scores, interpreted as measures of atrophy. The algorithm thus infers distinct spatiotemporal trajectories of regional volume loss, reflecting heterogeneous patterns of ALS neurodegeneration. The present analysis employed the Python implementation of SuStaIn (Aksman et al., 2021).

To ensure stable SuStaIn modeling with our sample size, we selected 14 regions from the 62 previously defined ROIs. The selection was primarily guided by the canonical pTDP-43 neuropathological staging scheme described by Brettschneider et al. (2013), which proposes sequential involvement of the motor cortex, corticospinal pathways, brainstem nuclei, frontal cortex, striatum, and medial temporal structures in ALS progression. Accordingly, we included regions corresponding to early motor system involvement (precentral and paracentral gyri, corticospinal tract, and brainstem), followed by frontal regions implicated in stage-2 pathology (superior frontal and rostral middle frontal cortices), striatal structures associated with later stages (caudate and putamen), and medial temporal regions involved in advanced stages (hippocampus, entorhinal, parahippocampal cortex, and amygdala). To complement these neuropathologically defined regions, we also included the superior and inferior parietal cortices. While not part of the canonical Brettschneider staging scheme, these regions have been consistently identified in structural MRI studies as key nodes of extra-motor involvement in ALS, exhibiting significant cortical thinning and volume loss, as well as alterations in structural brain networks (Agosta et al., 2012; Fortanier et al., 2019; Mezzapesa et al., 2013; Verstraete et al., 2012). This ROI set captures the major anatomical systems implicated in ALS pathology while limiting the number of biomarkers to maintain statistical stability and interpretability in SuStaIn modeling. For each ROI, 2 w-score abnormality thresholds (1 and 2) were used, yielding 28 model events. The maximum w-score threshold per region was set at 2 or 3 depending on the regional w-score distribution.

Model fitting was performed on baseline data, with eightfold cross-validation used to determine the optimal number of subtypes (*k*). Model selection was guided by the Cross-Validation Information Criterion (CVIC), which balances model fit and complexity (lower values indicate better fit), and the out-of-sample log-likelihood, assessing predictive generalization (higher values indicate better fit). Model uncertainty was estimated through 10,000 Markov Chain Monte Carlo (MCMC) iterations.

A single-subtype SuStaIn model (*k* = 1) was fitted using the same input parameters to provide a continuous, unified stage estimate across all patients. This cohort-level progression trajectory was primarily used to benchmark the model’s overall staging sequence against the established Brettschneider pathological stages (Brettschneider et al., 2013).

Participant-level subtype and stage assignments were obtained by maximizing the posterior likelihood of stage given the participant’s regional biomarker profile within each subtype. Individuals assigned to stage 0, indicating no abnormal regional w-scores, were classified as Normal Appearing (S0), while those with stage > 0 were assigned to their most probable subtype (Young et al., 2018).

### 2.5 Longitudinal consistency

The trained SuStaIn model derived from baseline data was subsequently applied to the longitudinal MRI scans to infer subtype and stage assignments at each available follow-up visit. This allowed assessment of the temporal consistency of the model’s subtype and stage predictions within individuals over time.

Subtype assignment was defined as *consistent* if participants either (i) remained assigned to the same subtype across visits or (ii) progressed from the Normal Appearing group (stage 0, i.e., no detectable atrophy at baseline) to any SuStaIn-defined subtype. Conversely, changes from one established subtype to another (e.g., S1 → S3) were considered *inconsistent*.

Stage evolution was defined as *consistent* if a participant remained at the same stage or progressed to a later stage at follow-up, reflecting monotonic disease advancement. Regression to an earlier stage was classified as *inconsistent*.

To evaluate assignment confidence, subtype and stage probabilities at the earliest visit were compared between *consistent* and *inconsistent* cases using a Student’s t-test for normally distributed data or a Mann–Whitney U test otherwise.

To ensure consistent and interpretable subtype characterization for group-level analyses, patients exhibiting longitudinal subtype transitions were assigned a single, most confident subtype. Specifically, for individuals whose inferred subtype changed across visits, the subtype with the highest posterior assignment probability across all time points, excluding S0 (Normal Appearing), was selected as their final representative subtype. Importantly, disease stage values were retained across visits to accurately reflect their individual disease progression timeline. For example, participants moving from S0 to S1 would retain their previous stage (stage=0), thereby maintaining information about the individual’s prior S0 status while allowing for a coherent subtype classification. This approach maintains the temporal integrity of SuStaIn-derived stages while ensuring that each participant contributes a single, most probable subtype to downstream clinical and imaging analyses, thereby improving model consistency and biological interpretability.

### 2.6 Statistical analyses

#### 2.6.1 Cross-sectional and longitudinal group comparisons

Group comparisons of longitudinal continuous clinical features were performed using a linear mixed-effects (LME) model applied to the longitudinal data, specified as follows:

$$\begin{array}{l} features\;∼1\;+\;Dx\;+\;\Delta Time\;+\;Dx\;:\;\Delta Time\;+\;Age_{bl}\;+\;Sex\;\\ \;\;+\;(1\;|\;ID), \end{array}$$ (1)

where Dx is a categorical fixed effect contrasting groups (ALS vs controls, or SuStaIn-inferred ALS subtypes) to assess group-level and longitudinal differences. The Dx:ΔTime interaction term captures group differences in the rate (slope) of longitudinal change. Age_bl_ represents age at baseline (first visit), and sex is a categorical covariate contrasting males and females. Participant ID was included as a random intercept to account for within-subject correlations across repeated measures.

For comparisons among ALS subtypes, the model additionally controlled for the confounding effect of disease progression (i.e., differences basine atrophy levels) by including the inferred disease stage at baseline (Stage_bl_) as a covariate. This ensures we isolate regional atrophy differences that are uniquely linked to the subtypes themselves.

Group differences in baseline demographic and clinical variables (e.g., age, sex, years of education, site of onset, ECAS, ALSFRS-R, DPR) were assessed using regression models appropriate to variable type. Continuous variables were analyzed using ordinary least squares (OLS) regression, binary variables using logistic regression, and ordered categorical variables using an ordinal logistic regression model.

For imaging analyses, regional w-scores were compared using an analogous LME model with the following specification:

$$\begin{array}{l} regional\_wscore\;∼1\;+\;Dx\;+\;\Delta Time\;+\;Dx\;:\;\Delta Time\\ \;\;+\;(1\;|\;ID).\; \end{array}$$ (2)

In this case, age_bl_, sex, and scanner effects had already been accounted for during the computation of the w-scores. The same modeling framework was applied for comparisons among ALS subtypes, including Stage_bl_ as an additional covariate.

For each set of group comparisons (e.g., S1 vs. S2, ALS vs. HCs), correction for multiple comparisons was performed by controlling the false discovery rate (FDR) at a level of *p* < 0.05. This correction was applied independently across two distinct sets of features: one for all clinical measures and the other for all imaging measures.

#### 2.6.2 Survival analysis

Survival outcome was defined as the composite end point of death, tracheostomy, or assisted ventilation for a minimum of 22 hours per day. Patients were censored if they were alive 5 years after the baseline MRI visit, lost to follow-up, or if they opted for medical assistance in dying (MAID). The final survival analysis cohort included 185 out of 198 patients, all of whom had follow-up data after the initial MRI visit, resulting in a censoring rate of 56%.

A Kaplan–Meier (KM) model was employed to visually represent the survival probabilities over time for each of the inferred ALS subtypes. The statistical significance of the difference in survival distributions between these subtypes was formally determined using the log-rank test.

#### 2.6.3 Association analysis: Clinical and imaging features with disease stages

In addition to conventional longitudinal modeling of atrophy progression over time (Dx:Δtime), we performed association analyses between SuStaIn-inferred disease stage and both imaging and clinical features. While longitudinal LME models capture linear, time-dependent change, the stage-based approach evaluates disease-linked progression, that is, how strongly each feature co-varies with the probabilistic disease stage inferred by the SuStaIn model. By including ΔTime as a covariate, this analysis isolates disease-related variance from simple effects of elapsed time, allowing us to identify brain regions and clinical measures most tightly coupled to disease advancement along each subtype’s inferred trajectory. Together, the stage- and time-based analyses provide complementary perspectives on ALS progression and serve as a validation of the biological and clinical relevance of the SuStaIn staging model.

The association between features and disease stages was assessed within each subtype, and subsequently across all ALS patients combined (including the S0 group). Baseline demographic and clinical variables were analyzed using OLS regression, with stage as the primary predictor. Longitudinal clinical features were assessed using an LME model with stage as the primary predictor, ΔTime as a fixed effect to account for the natural and linear effect of elapsed time, and participant ID as a random intercept to account for repeated measures. For both baseline and longitudinal clinical analyses, age_bl_ and sex were included as covariates when appropriate, with years of education additionally included for models involving the continuous raw ECAS cognitive metrics.

Similarly, the association with longitudinal imaging features (w-scores) was also assessed using an LME model with stage and ΔTime as fixed effect and ID as a random intercept. Notably, age_bl_ and sex were omitted from the imaging LME model, as their effects had already been accounted for during the w-score normalization process.

Correction for multiple comparisons was applied to the entire set of association tests. This was achieved by controlling the FDR across all calculated *p*-values (for both clinical and imaging associations) at a level of *p* < 0.05.

## 3 Results

The results are organized to reflect the analytical workflow. We first present the SuStaIn modeling outputs, including the single-subtype solution, the selection of the optimal number of subtypes, and the positional variance diagrams (PVDs) illustrating the model-inferred sequence of regional changes for the three-subtype solution. We then assess the longitudinal consistency of subtype and stage assignments across visits, which determines the most confident subtype classification for each participant. These final assignments are subsequently used to characterize the clinical and imaging properties of the subtypes, including cross-sectional and longitudinal volumetric patterns derived from linear mixed-effects models applied across the whole brain, encompassing gray matter, white matter tracts, and ventricles. While broad concordance with the model-inferred sequences depicted in the PVDs is expected, these whole-brain analyses extend beyond the 14 regions used as input to the SuStaIn model, further quantifying the magnitude and spatial extent of atrophy and expansion patterns across the full brain. Finally, we examine the association between model-inferred disease stages and both clinical and imaging measures, accounting for elapsed time.

### 3.1 Cohort characteristics

One hundred forty-four HCs and 198 patients with ALS were included in this study. A summary of the participants’ demographics, cognitive, and functional performance is presented in Table 1. Mean age in the patients with ALS was significantly greater than that of HCs. There was a greater proportion of men in the patients with ALS than the HCs. Years of education were slightly lower in the patients with ALS than the HCs. As expected, the patients with ALS showed greater cognitive and functional impairment. Significant differences were found in finger and foot tapping, and ECAS scores.

**Table 1.** Baseline cohorts demographics.

	HCs	Patients with ALS	*p* value
*N* visit (v1/v2/v3)	144/110/84	198/120/80	0.16^a^
Age, yr	57 (40-77)	60 (40-86)	<0.01^b^
Sex, M:F	62:82	128:70	<0.01^b^
Years education, yr	16 (7-28)	15 (4-28)	<0.05^b^
Finger tapping	57 (26-90)	41 (0-83)	<0.0001^c^
Foot tapping	41 (18-76)	24 (0-64)	<0.0001^c^
ECAS total score	116 (91-134)	108 (47-127)	<0.0001^b^

Values are expressed as median (range). Group comparisons were performed using:

a Ordinal logistic regression for ordered categorical variables.

b Ordinary least squares (OLS) regression for baseline continuous variables.

c Linear mixed-effects (LME) models for longitudinal continuous variables.

ALS = amyotrophic lateral sclerosis; ECAS = Edinburgh Cognitive and Behavioural ALS Screen.

### 3.2 SuStaIn input features and optimal number of subtypes

**Fig. 1.**
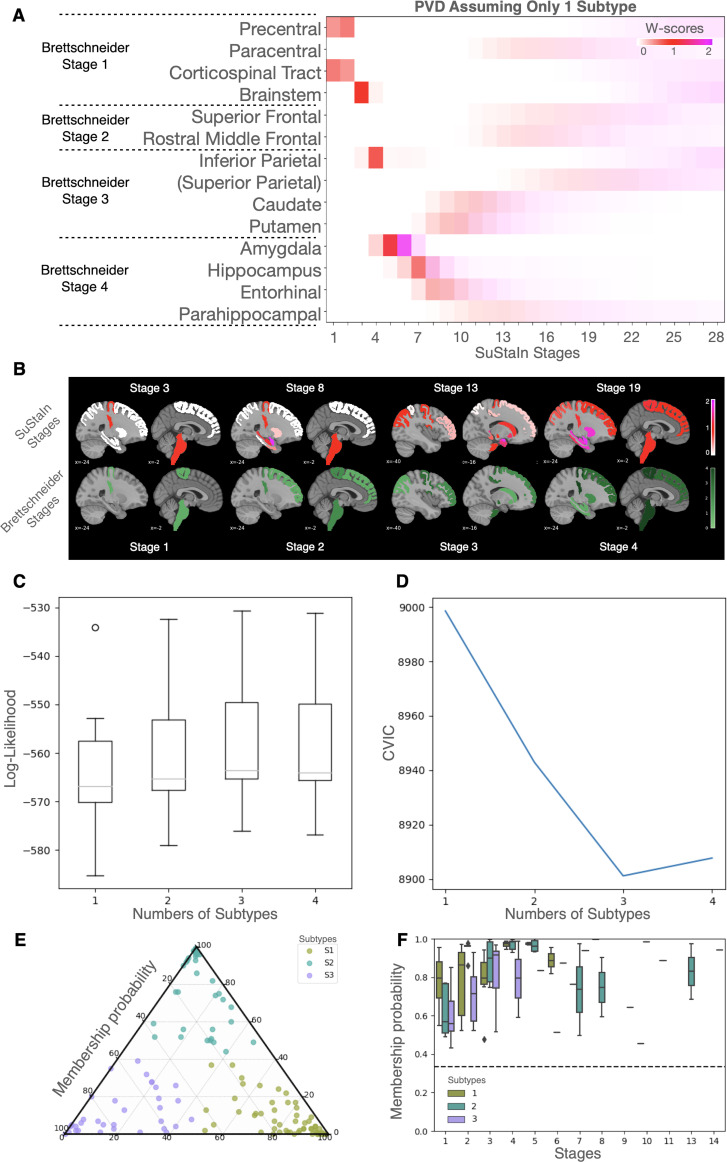
Subtype and stage inference modeling. (A) displays the positional variance diagrams (PVDs) obtained when the SuStaIn model was initially run assuming only one subtype. This PVD illustrates the single, assumed sequence of regional atrophy progression, with the X-axis representing the SuStaIn disease stage (1–28) and the Y-axis listing regions of interest (ROIs). Color intensity reflects the severity threshold reached (w-score=1 in red; =2 in magenta) and the model’s certainty (darker color = high certainty). (B) (Top) shows the spatial distribution of brain atrophy patterns across selected SuStaIn stages (3, 8, 13, 19), with the color bar indicating the cumulative probability of abnormality. (Bottom) depicts the corresponding canonical Brettschneider pathological stages (1–4) mapped anatomically for comparison. The color bar indicates the cumulative count of stages since a region has appeared. (C) and (D) show, for each subtype model (k = 1–4), the distribution of average negative log-likelihood (C), and the cross-validation information criterion (CVIC) (D), respectively. Higher log-likelihood and lower CVIC represent better model fit. (E) is a ternary plot that shows the membership probability for individual patients (dots) across the three inferred subtypes (S1, S2, S3). (F) displays the membership probability of assigned subtypes across the inferred disease stages. The dotted line represents the chance level (random assignment probability), which is confidently exceeded across all non-zero stages.

Figure 1A shows the SuStaIn-derived sequence of regional progression when restricting to a single ALS subtype. The w-score heatmap highlights early, pronounced involvement of the primary motor cortex (precentral, paracentral), corticospinal tract, and brainstem, which is consistent with Brettschneider’s stage 1 pathological spread. This is further illustrated in Figure 1B (top row), where SuStaIn stage 3 predominantly displays motor cortex and brainstem atrophy. As progression advances, the distribution expands to include parietal regions (stage 4), followed by medial temporal structures: amygdala, hippocampus, entorhinal, and parahippocampal cortices (between stages 4 and 13). Subcortical nuclei (caudate, putamen) emerge between stages 8 and 15, with association cortices (superior frontal, rostral middle frontal) affected at later stages (>13).

The bottom row in Figure 1B depicts the corresponding Brettschneider stages, mapped to canonical anatomical blocks: stage 1 aligns well with early motor and brainstem involvement; stage 2 localizes to superior and middle frontal regions; stage 3 encompasses parietal cortex and striatal nuclei; and stage 4 includes limbic temporal regions. When directly compared, the SuStaIn model recapitulates the major axis of ALS pathological progression described by Brettschneider but shows delayed involvement of prefrontal and striatal regions. These differences may reflect cohort variability and the sensitivity of MRI-based progression modeling to extra-motor and association cortical involvement.

Figure 1C and 1D shows the test set log-likelihood and the Cross Validation Information Criterion (CVIC), respectively, for determining the optimal number of ALS subtypes (*k*). An optimum is clearly obtained at *k* = 3 subtypes, indicated by a log-likelihood that stabilizes after increasing as *k* increases from 1 to 3, and the CVIC reaching its minimum value. Figure 1E illustrates the three subtypes membership probability for each patient, all stages combined, while Figure 1F shows the boxplots representing subtype membership probability according to the inferred disease stage. The subtype assignment probability remained significantly above the chance threshold (indicated by the dotted line) throughout all non-zero SuStaIn stages, demonstrating the model’s high confidence in its classification.

### 3.3 SuStaIn-inferred trajectories

**Fig. 2.**
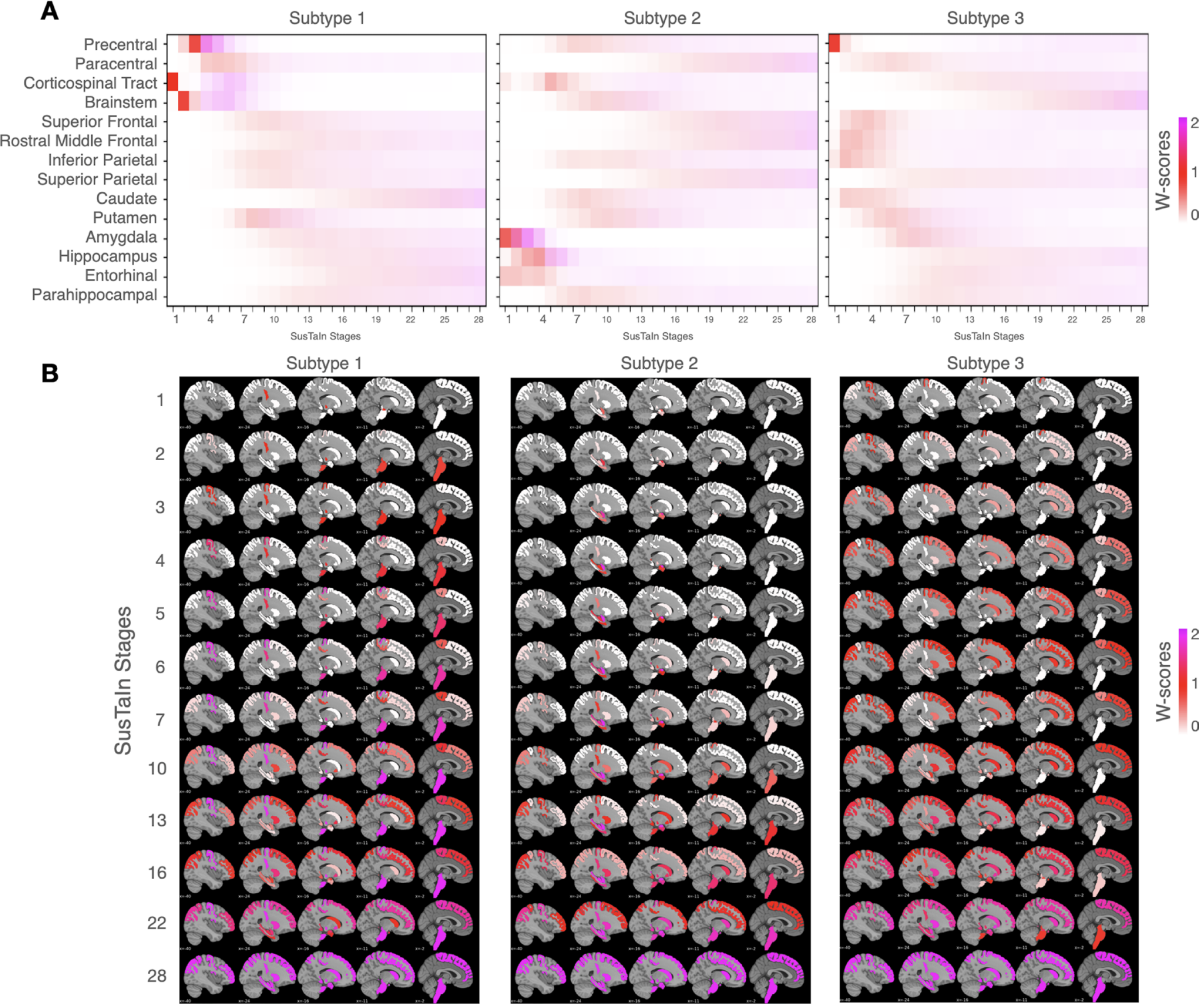
Progression patterns. Inferred trajectory of regional atrophy progression in subtypes. (A) displays the positional variance diagrams (PVDs), representing the inferred sequence and variability of regional atrophy progression for each subtype. The X-axis is the derived disease stage, while the Y-axis lists the specific ROIs. Each colored block indicates the stage at which an ROI crosses a pathological threshold, with the color corresponding to the severity score threshold reached (w-score = 1 in red; = 2 in magenta). The density of the color reflects the model certainty regarding that region’s position in the overall sequence, where darker colors indicate high certainty (low positional variance) and lighter colors indicate lower certainty. (B) presents the corresponding regional cumulative probability of abnormality overlaying brain sagittal views, illustrating the overall spatial pattern of atrophy at different stages of the inferred trajectory for each subtype.

Figure 2 illustrates the spatiotemporal progression of atrophy estimated by SuStaIn across the three ALS subtypes. Each panel shows the sequence in which regional deformation (W-score) abnormalities appear and spread across disease stages. Subtype 1 shows a motor-predominant pattern consistent with classical ALS progression. Early stages (Stages 1–6) reveal initial atrophy in the CST, followed by the brainstem, precentral, and paracentral gyri. As disease stages advance (from Stage 7), atrophy extends to frontoparietal and striatal structures (putamen), and finally involves limbic and medial temporal regions (amygdala, hippocampus, entorhinal, parahippocampal cortex) at later stages (after Stage 10), terminating with an atrophy in caudate, from Stage 16. Subtype 2 displays a limbic-onset pattern. The earliest stages (Stages 1–7) are characterized by marked atrophy in amygdala, entorhinal cortex, and hippocampus, indicating early limbic involvement. Atrophy of the CST emerges around Stage 5, followed by progressive extension to precentral gyrus, brainstem, inferior parietal, caudate, putamen, and parahippocampal regions. Later stages (after Stage 16) show additional frontal and parietal cortical involvement (superior frontal, rostral middle frontal, superior parietal), suggesting a spread from limbic to motor and associative cortices. Subtype 3 demonstrates a motor–frontostriatal pattern. The first affected regions include the precentral gyrus, followed by superior and inferior frontal and inferior parietal cortices, together with caudate at Stage 2. Progression continues with putaminal and amygdalar involvement, later reaching paracentral areas and ultimately the brainstem in advanced stages.

### 3.4 Longitudinal consistency

**Fig. 3.**
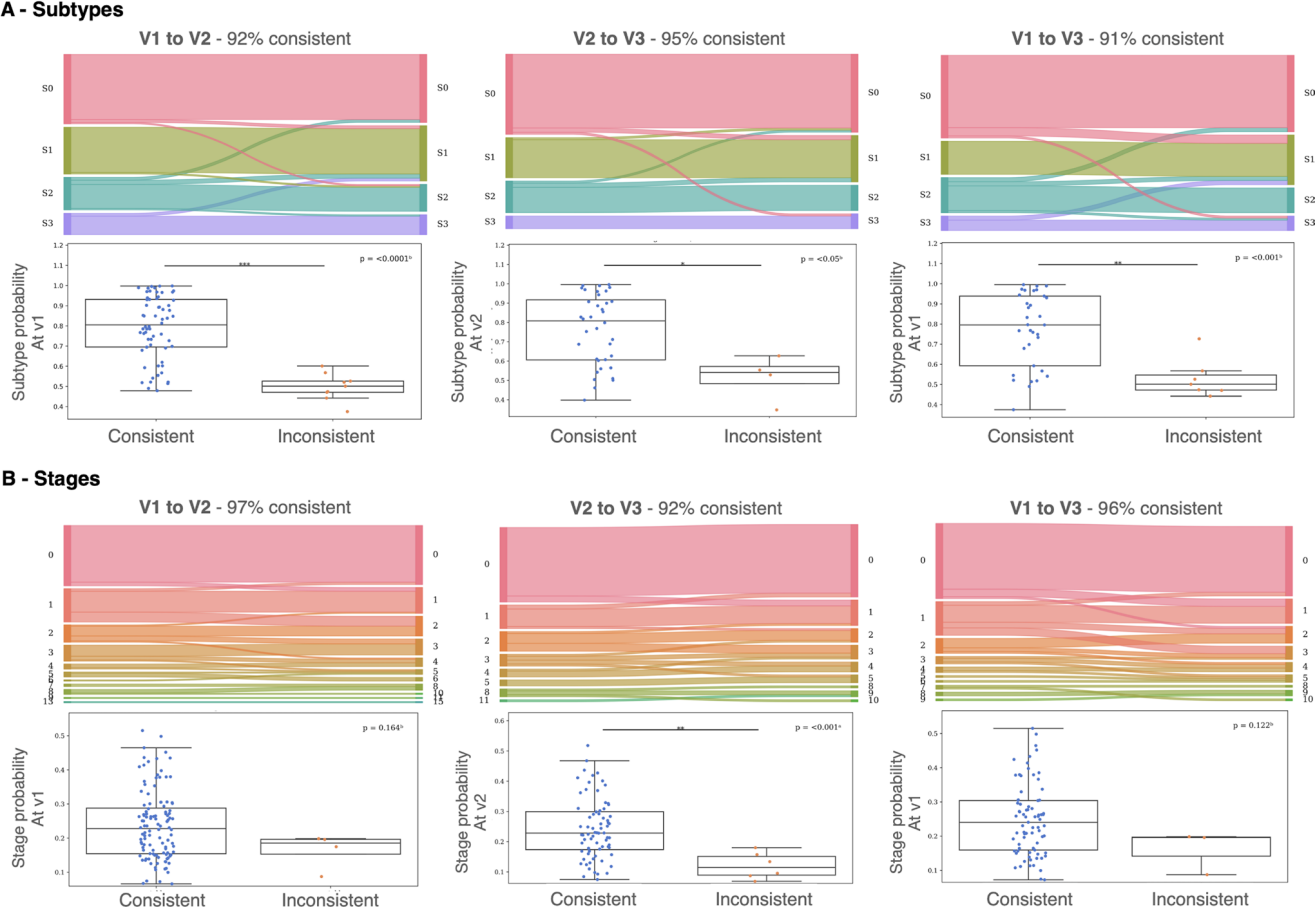
Longitudinal consistency of SuStaIn subtype and stage assignments. The Normal Appearing (S0) group was included, representing individuals assigned to Stage 0. Sankey diagrams (Top) and probability box plots (Bottom) illustrate the between-visit consistency of SuStaIn-inferred subtypes (A) and stages (B) across MRI time points: V1→V2 (left), V2→V3 (middle), and V1→V3 (right). In the Sankey diagrams, each horizontal block represents the total number of individuals assigned to a given subtype (or stage) at that visit. The connecting flows represent individual patient transitions between visits, with the width of each flow proportional to the number of individuals following that trajectory. The Sankey flows are color coded to represent the proportion of patients within each subtype or stage at the initial visit. Subtype and stage assignment probabilities from the earliest visit were compared between consistent and inconsistent cases using Student’s t-tests for normally distributed data or Mann–Whitney U tests otherwise. Statistical significance is denoted as follows: *p < 0.05, **p < 0.01, ***p < 0.001. Consistent cases were defined as those showing (i) no subtype or stage change, (ii) a transition from S0 to a subtype, or (iii) progression to a higher disease stage.

Figure 3 illustrates the longitudinal consistency of SuStaIn subtype and stage assignments across MRI visits using Sankey diagrams (top) and corresponding box plots of assignment probabilities (bottom). Subtype consistency (Fig. 3A) was high overall, with 92% consistency between V1 and V2, 95% between V2 and V3, and 91% between V1 and V3. Most participants either remained within the same subtype across visits or transitioned from S0 to a subtype, both patterns being considered consistent with expected disease evolution. This pattern indicates robust reproducibility of SuStaIn subtype classification over time. In contrast, cases that transitioned between subtypes (“inconsistent”) showed significantly lower subtype assignment probabilities than consistent cases across all intervals (*p* < 0.05 to *p* < 0.0001), suggesting that misclassification primarily occurred among participants with less confident baseline assignments. Stage consistency (Fig. 3B) was even higher, reaching 97% consistency from V1 to V2, 92% from V2 to V3, and 96% from V1 to V3. Similar to subtypes, inconsistent stage transitions (e.g., to a lower stage) were associated with lower assignment probabilities, although significance was reached only for the V2–V3 comparison (*p* < 0.001), with the remaining contrasts showing a non-significant trend toward lower probabilities in inconsistent cases.

Among patients with longitudinal data, 11 out of 198 showed a transition in their inferred subtype between visits. Accordingly, their most confident subtype (i.e., the subtype with the highest assignment probability across visits) was reassigned for subsequent clinical and imaging analyses. The transitions were distributed as follows: 5 from S0 to S1, 1 from S0 to S2, 1 from S0 to S3, 1 from S2 to S1, 1 from S2 to S3, and 2 from S3 to S1. This resulted in a final sample comprising 61 Normal Appearing (S0) individuals and 137 participants assigned to 1 of 3 SuStaIn-inferred ALS subtypes (S1 = 65, S2 = 39, S3 = 33).

### 3.5 Demographic and clinical subtypes comparisons

**Fig. 4.**
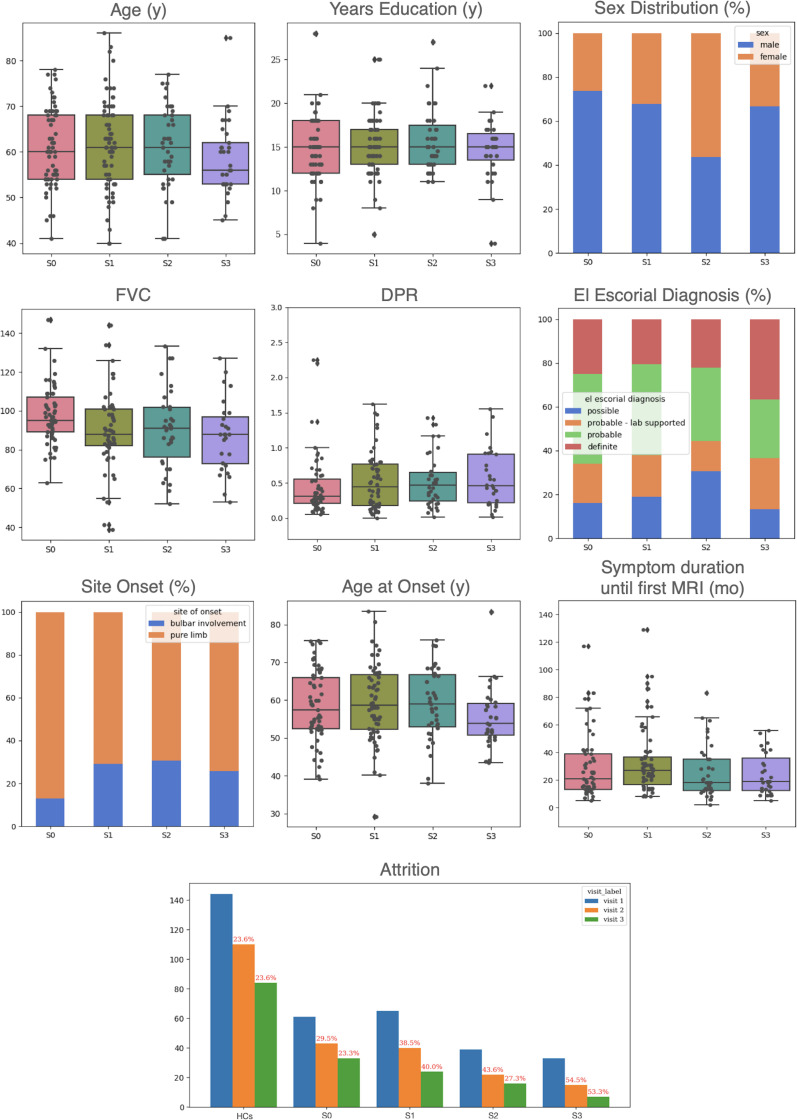
Baseline demographic and clinical features across SuStaIn-inferred ALS subtypes. The Normal Appearing (S0) group was included, representing individuals assigned to Stage 0. Ten comparative panels illustrate differences in age, education, sex, FVC, DPR, El Escorial diagnostic categories, site of onset, age at onset, symptom duration prior to MRI, and patient attrition among inferred ALS subtypes and S0 group. Box plots show medians, interquartile ranges, and outliers for continuous variables (age, education, FVC, DPR, age at onset, symptom duration); bar or stacked charts represent categorical variables (sex, El Escorial diagnosis, site of onset, attrition). Attrition panels indicate the number of patients per visit for each subtype and percentage lost per group in red (attrition includes censored and deceased patients). Group differences for continuous baseline variables were analyzed using OLS regression, while repeated longitudinal measures were assessed using LME models. Binary outcomes were evaluated via logistic regression and ordered categorical outcomes by ordinal logistic regression. Multiple comparisons were FDR corrected (*p* < 0.05). No differences between subtypes were statistically significant after correction. ALS = amyotrophic lateral sclerosis; DPR = disease progression rate, estimated using (48-ALSFRS-R)/symptom duration; FDR = false discovery rate; FVC = forced vital capacity; HCs = healthy controls; LME = linear mixed effects; MRI = magnetic resonance imaging; OLS = ordinary least squares.

Analysis of baseline demographics and clinical characteristics (Fig. 4) revealed distinct profiles across the SuStaIn-derived subtypes. While most subgroups displayed comparable symptom duration, DPR, and education levels, S3 patients were the youngest cohort, exhibiting the lowest median age at the first visit (56 years old) and median age at onset (54 years old), although these differences did not reach statistical significance compared with others. FVC appeared higher in S0 than in the subtype’s groups, but this difference also did not survive FDR correction. A major difference was observed in sex distribution: S2 exhibited a near-equal male-to-female ratio (17:22), suggesting a distinct female predominance compared with the predominantly male S0 (45:16), S1 (44:21), and S3 (22:11) cohorts. The El Escorial diagnosis also showed variation: S0 and S1 were most frequently diagnosed as “Probable”, while S3 was most frequently “Definite”. Notably, S2 had a high proportion of “Possible” and “Probable” diagnoses. All groups primarily presented with limb-only onset, with S0 showing the most substantial difference (53 limb-only vs 8 including bulbar). Finally, longitudinal attrition varied, with S1 maintaining the most comprehensive follow-up data (less than 30% attrition across three visits), whereas S3 suffered the highest percentage of attrition (over 50% loss between each visit), suggesting rapid progression or high burden impacting clinic attendance. None of the differences in features across subtypes were statistically significant.

**Fig. 5.**
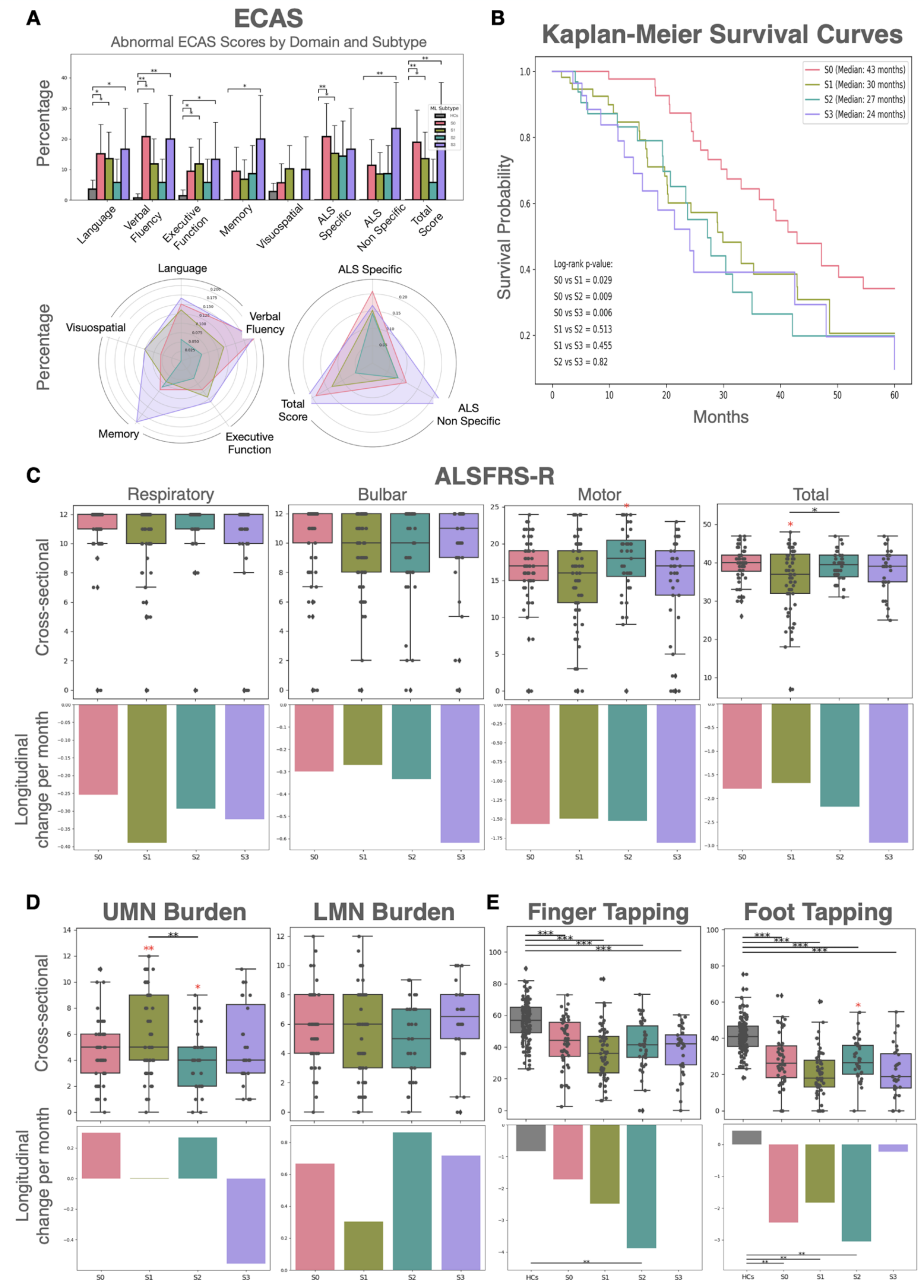
Cognitive, functional, motor, and survival differences across ALS subtypes. The Normal Appearing (S0) group was included, representing individuals assigned to Stage 0. Healthy controls (HCs) were also included when appropriate. (A) Bar graphs and radar plots illustrate abnormal ECAS scores by cognitive domain for ALS subtypes and HCs, including ALS specific (language, fluency, executive) and ALS non-specific (memory, visuospatial). (B) Kaplan–Meier survival curves compare probabilities and median survival durations for each subtype, with log-rank *p*-values indicated. (C) ALSFRS-R boxplots show cross-sectional respiratory, bulbar, motor, and total scores, while corresponding bar charts report each subtype’s mean longitudinal change per 4 months. (D) UMN and LMN burden scores are displayed using boxplots and mean longitudinal decline per 4 months bar charts per ALS subtype. (E) Finger and foot tapping performance for ALS subtypes and healthy controls are summarized by boxplots (cross-sectional) and bar charts (mean longitudinal change per 4 months). Significant group differences, with FDR correction at *p* < 0.05, are indicated by asterisks. Red asterisks denote a subtype’s difference compared with all other ALS subtypes combined. Statistical significance levels are represented as follows: *p < 0.05, **p < 0.01, ***p < 0.001. Boxplots display the median, interquartile range, and outlying values for each group. Bar charts depict the mean monthly change per group. Group differences for repeated longitudinal measures were assessed using LME models. Binary outcomes were evaluated via logistic regression, and log-rank tests for survival data. ALS = amyotrophic lateral sclerosis; ALSFRS-R = Amyotrophic Lateral Sclerosis Functional Rating Scale-Revised; ECAS = Edinburgh Cognitive and Behavioural ALS Screen; FDR = false discovery rate; LME = linear mixed effects; LMN = lower motor neuron; OLS = ordinary least squares; UMN = upper motor neuron.

Figure 5 illustrates differences in clinical and cognitive function among subtypes. ECAS scores (Fig. 5A top) revealed distinct cognitive profiles, with varying degrees of impairment across domains and subtypes. S0 and S3 are associated with the highest rates of abnormal scores, indicating pronounced multidomain dysfunction. Specifically, S3 shows the greatest impairment in memory, language, and fluency domains, while S0 exhibits deficits in language and fluency. In contrast, S1 generally manifests only mild or minimal abnormalities, and S2 stands out with the lowest levels of cognitive dysfunction across all measured domains. Although rates of abnormal scores differ by subtype, these between-ALS subtype differences did not reach statistical significance within the cohort. Instead, significant differences in cognitive impairment were consistently identified only when comparing certain ALS subtypes with healthy controls, and only in specific ECAS domains. Radar plots (Fig. 5A bottom) further illustrate these patterns, showing that subtype 3 encompasses more widespread and severe cognitive compromise, followed by subtype 0, whereas subtype 2 remains the most spared.

The Kaplan–Meier Survival Curves (Fig. 5B) revealed the worst prognosis for the ALS subtypes: S1 (median: 30 months) and S2 (median: 27 months), with S3 exhibiting the shortest median survival time of 24 months. This was significantly lower than in the S0 group, which had a median survival of 43 months. Analysis of the ALSFRS-R total score (Fig. 5C) showed S2 with the highest motor score when compared with all the other ALS patients, while S1 had the lowest ALSFRS total score. Longitudinal analysis of the average change per month in ALSFRS-R scores did not yield significant differences between the cohorts. In terms of cross-sectional clinical disease burden (Fig. 5D), S1 showed the highest UMN burden, significantly greater than all other subtypes, while S2 displayed the lowest UMN burden and a trend toward lower LMN burden. Although cross-sectional LMN burden did not significantly differ between subtypes, S0 displayed relatively low UMN burden while maintaining LMN scores comparable with the other ALS groups. Consistent with its preserved motor profile, functional tests (Fig. 5E) showed that S2 achieved the highest cross-sectional foot tapping score and a trend toward higher finger tapping performance, indicating the best-preserved distal limb function. Longitudinally, S2 showed a pronounced decrease in finger tapping score compared with HCs and S3, whereas S3 was the only subtype whose tapping scores remained largely stable over time, similar to controls.

### 3.6 Cross-sectional contrasts confirmed separable imaging signatures

**Fig. 6.**
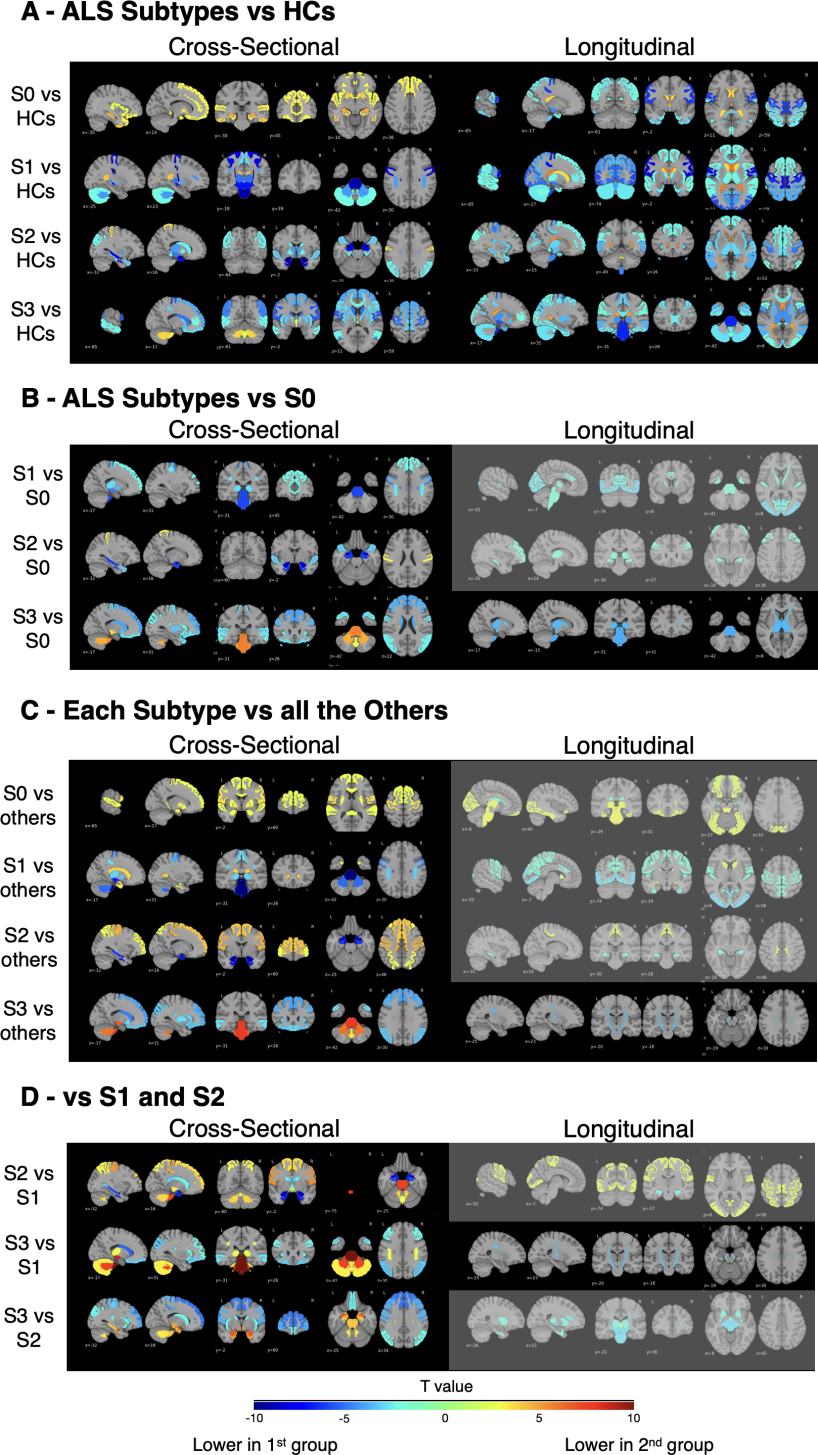
Maps of brain volume differences across SuStaIn-inferred subtypes, S0 and HCs using regional DBM w-scores. (A) Cross-sectional and longitudinal contrasts of S0 and each SuStaIn-inferred subtype (S1–S3) versus HCs. (B) Subtype comparisons relative to NA. (C) Each ALS subtype compared with all other subtypes. (D) Pairwise subtype contrasts (S2 vs S1; S3 vs S1; S3 vs S2). For each panel, *t*-value maps display regions with significant volume differences or ventricular expansion: positive *t*-values (yellow–red) reflect expansion or higher volume in the first group, while negative *t*-values (blue) indicate lower volume. Light gray overlays flag regions where contrasts did not survive FDR correction (*p* > 0.05). Group differences were assessed using LME models. Sample sizes: HC = 144, S0 = 61, S1 = 65, S2 = 39, S3 = 33. ALS = amyotrophic lateral sclerosis; HCs = healthy controls; LME = linear mixed effects.

Cross-sectional contrasts against healthy controls (Fig. 6A, left) revealed four separable imaging signatures.

The Normal Appearing (S0) group showed no regional atrophy versus controls (i.e., no negative t-values) and instead displayed positive t-values in limbic and basal nuclei (e.g., hippocampus *t* = 4.68, amygdala *t* = 4.07, putamen *t* = 3.92). Relative to all subtypes (Fig. 6C, left), S0 showed the greatest preservation across precentral, pallidal, accumbens, hippocampal, amygdalar, superior–temporal, putaminal, and superior–frontal regions, as well as the corpus callosum, consistent with a structurally intact, possibly compensatory, frontolimbic-striatal profile.

S1 exhibited the canonical motor/CST/brainstem atrophy pattern (e.g., corticospinal tract *t *= -9.69, brainstem *t *= -9.13, precentral *t *= -8.05) accompanied by ventricular enlargement (third *t* = 5.18; lateral *t* = 4.05). Compared with the S0 group (Fig. 6B, left), S1 showed extensive precentral and brainstem atrophy together with CST degeneration, consistent with early motor–system involvement. Relative to all other subtypes (Fig. 6C, left), S1 displayed relative sparing of the caudate, entorhinal cortex, and amygdala, indicating a selective motor-dominant trajectory with limited limbic participation.

S2 displayed a limbic-predominant pattern (amygdala *t* = -11.50, entorhinal *t* = -7.48, hippocampus *t* = -7.36), with a relatively spared postcentral gyrus (*t* = 3.49) and no ventricular enlargement. In comparison with the S0 group (Fig. 6B, left), S2 showed pronounced limbic and medial–temporal atrophy but maintained intact motor and parietal cortices. Relative to other subtypes (Fig. 6C, left), S2 showed preservation in superior and rostral-middle frontal, superior-parietal, postcentral, precentral, paracentral, and hand-knob regions, consistent with a limbic-onset trajectory that later extends toward motor networks.

S3 showed a striatal plus fronto-parietal/motor profile (e.g., precentral *t* = -5.72, caudate *t* = -5.23, inferior parietal *t* = -4.89) with enlargement of the third and lateral ventricles. Compared with the S0 group (Fig. 6B, left), S3 demonstrated extensive fronto-striatal and parietal involvement (superior-frontal *t* = -4.16, rostral-middle-frontal *t* = -3.90, putamen *t* = -3.59, caudate *t* = -4.17) but relative preservation of the brainstem, cerebellum, and CST. In the subtype-to-subtype contrasts (Fig. 6C, left), S3 exhibited relative preservation or enlargement of the brainstem, cerebellar white matter, ventral diencephalon, CST, and vermal lobules I-V and VIII-X, reflecting a fronto-striatal-dominant phenotype with maintained integrity of cerebellar–brainstem structures.

These separations were strongly validated by pairwise subtype contrasts (Fig. 6D, left): the S2 vs. S1 comparison highlighted the prominent limbic profile, while the S3 vs. S1 contrast emphasized the distinct striatal/fronto-parietal signature over the canonical motor pattern. Finally, the S3 vs. S2 contrast clearly demarcated the frontal-motor burden of S3 against the limbic atrophy characteristic of S2.

### 3.7 Longitudinal contrasts revealed separable imaging signatures

The longitudinal analysis, which measures the rate of atrophy change over time, provided a dynamic view of disease progression, often revealing aggressive spread that was not yet the dominant feature in the cross-sectional burden. Note that no longitudinal information was used in deriving the subtypes.

Despite lacking baseline atrophy, the S0 group demonstrates clear progressive motor decline versus controls (Fig. 6A, right), with the fastest rates of tissue loss observed in the precentral gyrus (*t* = -6.35), paracentral lobule (*t *= -6.31), and hand-knob (*t *= -5.38) regions. This suggests that while S0 is structurally intact at baseline, the pathology is already accelerating within the motor system. This atrophy is accompanied by a pronounced rate of ventricular enlargement (third ventricle *t* = 4.86, fourth ventricle *t* = 4.34, and lateral ventricle *t* = 3.71). S0 differences compared with all subtypes did not survive FDR correction and are overlaid with light gray (Fig. 6C, right).

Compared with controls (Fig. 6A, right), S1 exhibited a high rate of atrophy progression concentrated in the primary motor system. The highest observed atrophy rate was in the precentral gyrus (*t *= -8.14). This atrophy was accompanied by further expansion in the lateral ventricle (*t* = 5.57), inferior lateral ventricle (*t* = 5.38), third ventricle (*t* = 5.21), and fourth ventricle (*t* = 3.45). Intriguingly, the caudate showed less atrophy progression than in healthy controls (*t* = 3.56), indicating a relative sparing of this subcortical structure despite the aggressive motor pathway involvement. Other longitudinal S1 comparison contrasts did not survive FDR correction (Fig. 6B, C, D, right), except for the S3 vs S1 comparison (Fig. 6D, right), which is detailed below.

While cross-sectionally defined by limbic atrophy, S2 demonstrated a mixed longitudinal progression versus controls (Fig. 6A, right), with dominant motor-system decline (e.g., hand-knob *t *= -5.08, precentral gyrus (*t *= -4.48), and the brainstem (*t *= -4.77)) accompanied by limbic atrophy. This atrophy was accompanied by a pronounced rate of ventricular enlargement (lateral ventricle *t* = 5.33, third ventricle *t* = 5.14, inferior lateral ventricle *t* = 3.42, and fourth ventricle *t* = 3.15). Other contrasts involving S2 did not survive multiple-comparison correction (light gray overlay in Fig. 6B, C, D, right).

S3 progression rate compared with controls (Fig. 6A, right) was dominated by caudal and descending motor structures atrophy. The highest rate of atrophy was observed in the brainstem (*t *= -6.75), followed by the ventral diencephalon (*t *= -5.85), CST (*t *= -5.26), anterior thalamic rad (*t *= -4.72), precentral gyrus (*t *= -4.63), and thalamus (*t *= -4.57). S3 showed significant differences that survived FDR correction when compared with other ALS groups. Specifically, S3 demonstrated a significantly faster progression of atrophy in the brainstem (*t *= -4.01), thalamus (*t *= -3.81), CST (*t *= -3.74), and anterior thalamic radiations (*t *= -3.67) than S0 (Fig. 6B, right). Furthermore, S3 showed a unique acceleration of CST atrophy compared with S1 alone (Fig. 6D, right) and when compared with all other subtypes combined (Fig. 6C, right). This robust finding confirms that S3 is defined by a uniquely aggressive rate of degeneration along the central motor and thalamic-frontal pathways. This atrophy was accompanied by a severe rate of expansion in the third ventricle (*t* = 6.17) and lateral ventricle (*t* = 4.96).

### 3.8 Association analysis: Clinical and imaging features with disease stages

**Fig. 7.**
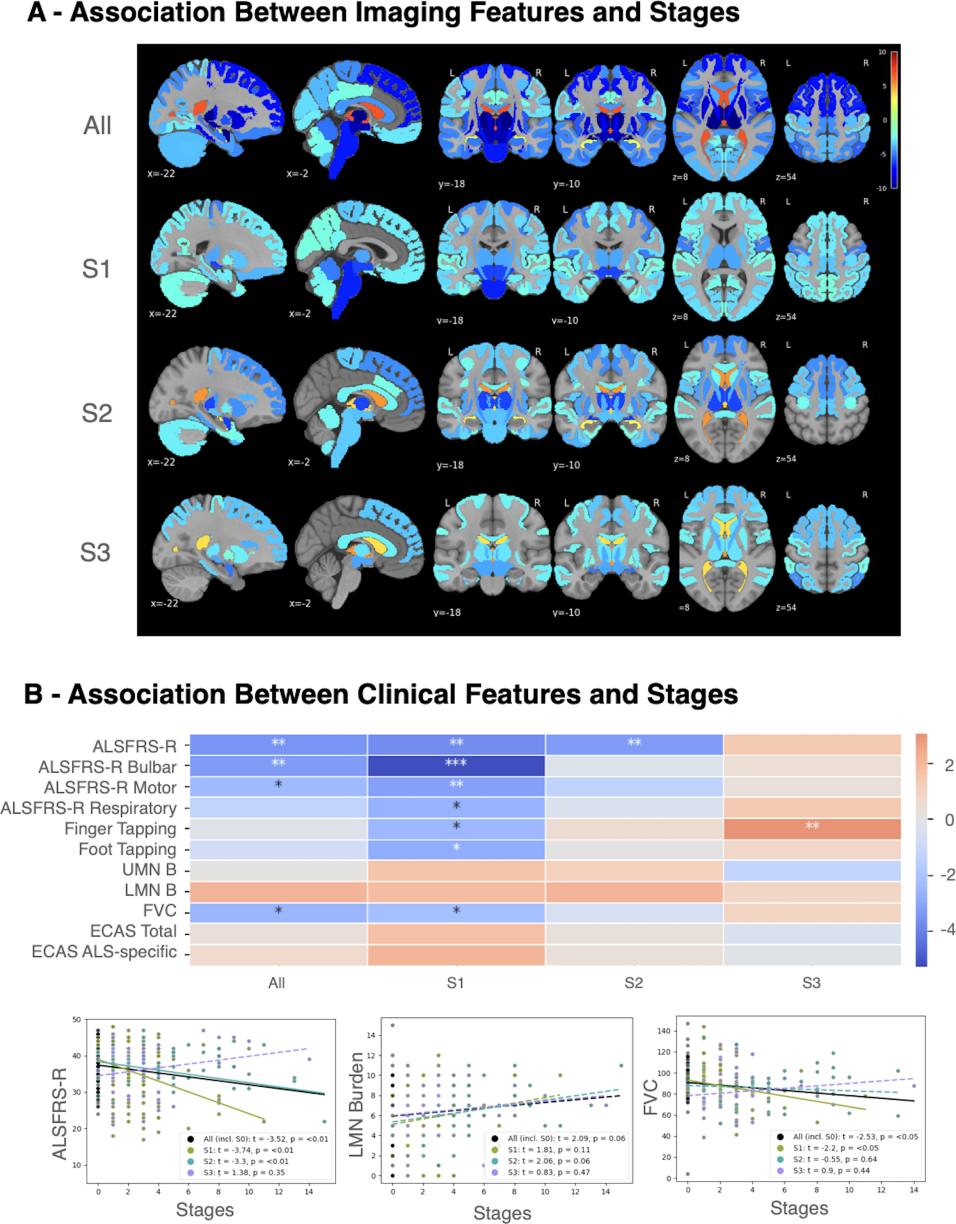
Associations between SuStaIn-inferred disease stage and longitudinal imaging or clinical features in ALS. (A) Brain maps display mixed-effects model associations (*t*-values, significant at *p* < 0.05 after FDR correction) between SuStaIn stage and regional longitudinal imaging features (w-scores) for the overall ALS cohort including S0 (“All”) and by inferred subtype (S1-S3). Blue shades indicate negative associations (advanced stage = greater atrophy), while red hues indicate positive associations (advanced stage = relative expansion, most often ventricular). (B) (Top) Heatmap summarizing mixed-effects model associations (t-values, FDR-corrected) between SuStaIn stage and key clinical variables across subtypes. Negative values (blue) indicate functional decline with advancing stage, while for UMN/LMN burden, red shades denote that higher stage is related to increased motor burden. The positive association observed for S3 finger tapping is driven by data sparsity and highly variable values at advanced stages, necessitating caution in interpretation. Asterisks indicate FDR-corrected statistical significance: *p < 0.05, **p < 0.01, ***p < 0.001. (Bottom) Representative scatterplots show stage associations with ALSFRS-R total score, LMN burden, and FVC. Colored lines (dashed when *p* > 0.05) represent subtype-specific fits, with black lines indicating the combined cohort, including the S0 group. ALS = amyotrophic lateral sclerosis; ALSFRS-R = Amyotrophic Lateral Sclerosis Functional Rating Scale-Revised; FDR = false discovery rate; FVC = forced vital capacity; LMN = lower motor neuron; UMN = upper motor neuron.

Widespread associations between SuStaIn stage and regional atrophy (w-scores) were observed across subtypes (Fig. 7A). At the group level, combining all ALS cases (including S0), the strongest negative associations with disease stage, indicative of progressive tissue loss, were found in limbic and subcortical regions. The most significant atrophy was recorded in the amygdala (*t *= -10.45), thalamus (*t *= -9.82), ventral diencephalon (*t *= -9.07), accumbens area (*t *= -8.49), and then putamen (*t *= -8.37), followed by parahippocampal, precentral gyrus, and frontal regions. Conversely, ventricular volumes (lateral: *t* = 6.76; third: *t* = 6.74; inferior lateral: *t* = 2.61) showed significant positive associations, reflecting the typical ventricular expansion that accompanies cerebral atrophy. Each subtype presented a distinct progression profile. S1 strongly supported a predominantly motor–subcortical trajectory, showing the tightest correlations between stage and atrophy in the brainstem (*t *= -6.82), ventral diencephalon (*t *= -6.12), precentral gyrus (*t *= -4.96), thalamus (*t *= -4.27), and the CST (*t *= -4.16). S2 exhibited robust associations in limbic–thalamic structures such as the amygdala (*t *= -6.29), thalamus (*t *= -6.16), and accumbens (*t *= -6.09), but also involved the inferior fronto-occipital fasciculus (*t *= -4.77), superior frontal (*t *= -4.69), putamen (*t *= -4.66), and CST (*t *= -4.59), indicating that while limbic regions are primary, motor and thalamic structures become substantially involved later. S2 also showed significant ventricular expansion (lateral: *t* = 5.19; third: *t* = 3.75). Subtype 3 (S3) was characterized by the most extensive cortical–subcortical involvement, with significant atrophy in the amygdala (*t *= -5.20), inferior parietal (*t *= -4.62), ventral diencephalon (*t *= -4.24), anterior thalamic radiations (*t *= -4.09), and frontal regions (caudal/rostral middle frontal, superior frontal: *t *= -3.65). Like the S2, ventricular expansion was positively correlated with stage (third: *t* = 5.18; lateral: *t* = 3.54).

Across clinical measures (Fig. 7B), SuStaIn stage showed expected negative associations with functional decline. ALSFRS-R and FVC both decreased significantly with increasing stage when analyzed across all subtypes, with the strongest effects observed in the Motor/CST-Dominant (S1) subtype (*p* < 0.05). Motor performance, assessed by finger and foot tapping frequency, showed a significant stage-dependent decline in S1. However, the unexpected increase in finger tapping metrics seen in S3 at later stages is likely an artifact of limited longitudinal data (Supplementary Fig. S1). With only five observations available at Stage ≥ 7 (versus 50 at Stage < 7), the model is highly sensitive to individual outliers, leading to numerical instability in the estimated trajectory for these later stages. LMN burden tended to increase with stage (*p* ≈ 0.06 after FDR), particularly in the combined and S2 groups, consistent with progressive lower motor neuron involvement. In contrast, UMN burden and ECAS scores showed no significant association. Representative scatterplots for ALSFRS-R, FVC, and LMN burden are shown in Figure 7, with the full association map presented in Supplementary Figure S1.

## 4 Discussion

In the present study, we applied the SuStaIn algorithm to DBM-derived regional brain volumes from CALSNIC-1 and CALSNIC-2 cohorts to identify ALS subtypes. In addition to one “Normal Appearing/Motor-Onset” (S0) group, SuStaIn modeling of cross-sectional DBM brain imaging revealed three biologically plausible ALS trajectories that align with and extend our cross-sectional and longitudinal analyses. By combining a probabilistic disease progression model with longitudinal mixed-effects validation, this work provides evidence that ALS subtypes can be reproducibly staged and clinically interpreted. Subtype and stage consistency across visits (≥90%) confirmed the biological validity of the inferred trajectories and supports their potential for longitudinal patient stratification.

In our cohort, the SuStaIn-inferred Normal Appearing (S0) group and disease subtypes demonstrated distinct imaging and clinical profiles, suggesting graded patterns of extra-motor involvement across the ALS-FTD spectrum (Strong et al., 2017).

### 4.1 S0 (“Normal Appearing/Motor-Onset”)

Appeared structurally intact at baseline, showing no cross-sectional atrophy relative to healthy controls and even relative hypertrophy in limbic and basal nuclei. This pattern may reflect early compensatory reorganization or neuronal dysfunction preceding detectable macrostructural atrophy, consistent with previous findings in ALS and related disorders (Abidi et al., 2020; Trubshaw et al., 2024). Despite this preservation, longitudinal analysis revealed clear motor–system decline, accompanied by pronounced ventricular expansion, suggesting delayed but eventual motor involvement. Clinical measures indicated relatively low UMN burden while LMN burden was comparable with other ALS subtypes, suggesting that early LMN pathology in the spinal cord and peripheral motor pathways may contribute to motor dysfunction before detectable cerebral structural changes emerge. Separately, early language and verbal fluency deficits despite imaging preservation suggest subtle frontotemporal network dysfunction that may precede detectable structural degeneration within the ALS-FTD spectrum. Consistent with this relatively preserved structural profile, individuals in S0 also showed the longest survival among the identified subtypes (~43 months).

### 4.2 Subtype 1 (“Motor/CST-Dominant”)

Exhibited the canonical ALS trajectory, beginning with corticospinal, precentral, paracentral, and brainstem atrophy, later spreading into frontoparietal and subcortical regions. These findings were supported by LME analyses showing prominent motor–system atrophy and progressive decline in motor and brainstem regions, along with extension in ventricles and caudate sparing compared with controls. When compared with S3, S1 exhibited a lower progression of CST atrophy. Stage–feature associations revealed strong stage dependence in the CST, thalamus, and brainstem, closely paralleling ALSFRS-R decline, confirming that SuStaIn staging captures biologically and clinically meaningful motor progression. Clinically, individuals in S1 showed classic ALS features, including higher UMN burden, ALSFRS-R decline, and intermediate survival (~30 months), with mild cognitive dysfunction, consistent with relative preservation of frontal–subcortical networks compared with more severe phenotypes on the ALS-FTD spectrum.

### 4.3 Subtype 2 (“Limbic-Onset with Motor Progression”)

Followed a distinct trajectory characterized by early atrophy in limbic and medial temporal regions (amygdala, hippocampus, entorhinal cortex), later extending into motor and associative cortices. Cross-sectionally, S2 showed the most pronounced limbic atrophy; longitudinally, it evolved toward a mixed limbic–motor profile with ventricles extension when compared with HC. Other longitudinal contrasts involving S2 did not survive multiple-comparison correction. Stage correlations confirmed this shift, showing limbic dominance cross-sectionally and emerging CST/paracentral associations longitudinally, consistent with a transition from limbic to motor involvement. Clinically, S2 featured balanced sex distribution, lower UMN burden, higher ALSFRS-R motor scores, more “Possible” ALS diagnoses, intermediate survival (~27 months), and cognition comparable with controls. This relative preservation of executive function corresponded with structural preservation of frontal regions (superior and rostral middle frontal cortices and superior parietal areas; Fig. 6C), despite prominent limbic atrophy.

### 4.4 Subtype 3 (“Striatal-Fronto-parietal with Motor-Thalamic Rapid Progressor”)

Displayed the most extensive and aggressive neurodegeneration. Early atrophy appeared in precentral and caudate regions, spreading to frontoparietal and striatal networks and later to limbic and motor-associative areas. Cross-sectional LME analysis confirmed dominant fronto-parietal and striatal atrophy with relative preservation of cerebellar white matter. Longitudinally, S3 was the only subtype to have a difference in progression of atrophy when compared with other subgroups. Specifically, S3 exhibited a markedly faster progression of atrophy in the brainstem, thalamus, CST, and anterior thalamic radiations than S0, consistent with an aggressive motor–thalamic trajectory. This accelerated rate was further highlighted by the fact that CST atrophy in S3 had a significantly higher progression rate than S1 alone and than all other combined subgroups. Stage–feature associations showed that S3 progression was driven by amygdala, fronto-parietal, striatal, and thalamic correlations, supporting a frontal–subcortical network trajectory rather than isolated motor tract degeneration. Notably, CST and brainstem atrophy were time dependent but not stage dependent, suggesting rapid continuous decline decoupled from SuStaIn staging once ΔTime was controlled. This distinction highlights how integrating SuStaIn stage with longitudinal modeling differentiates true disease staging effects from simple temporal change. Anatomically, the combination of striatal-fronto-parietal degeneration and rapid motor–thalamic progression overlaps with neural networks known to be particularly vulnerable in ALS-FTD. While individuals with clinical dementia were excluded, S3 likely captures the segment of the ALS-FTD spectrum with the highest extra-motor burden. Clinically, S3 showed the most severe multidomain cognitive impairment (executive dysfunction, memory, language, and verbal fluency) and the poorest survival (~24 months), consistent with disruption of frontal–subcortical circuits frequently implicated in the progression toward ALS-FTD.

Our findings align with and extend previous imaging-based ALS stratification efforts. Baumeister et al. (2025), using CALSNIC and Utrecht data, applied the method of contrastive trajectory inference and identified three ALS subtrajectories: ALS_motor, ALS_diffuse, and ALS_limbic, based on gray matter density and white matter fractional anisotropy. Their ALS_motor and ALS_limbic clusters closely correspond to our S1 (“Motor/CST-Dominant”) and S2 (“Limbic-Onset with Motor Progression”) subtypes, both defined by predominant motor and limbic degeneration, respectively. Our third subtype (S3) extends this framework by revealing extensive fronto-striatal and parietal atrophy at baseline and a uniquely aggressive rate of longitudinal degeneration along motor–thalamic pathways. Unlike Baumeister et al. (2025), who analyzed cross-sectional data, we incorporated longitudinal validation, ΔTime-adjusted mixed-effects modeling to compare longitudinal change between groups and link SuStaIn-inferred stage with both clinical decline and DBM progression, distinguishing disease-stage effects from simple temporal change.

Our findings also extend the work of Shen et al. (2023) who applied SuStaIn to volumetric MRI data from ALS, ALS-FTD, and behavioral variant frontotemporal dementia (bvFTD) cohorts, identifying one Normal Appearing group and two atrophy subtypes: prefrontal/somatomotor-predominant and limbic-predominant. Their prefrontal/somatomotor subtype parallels our “Motor/CST-Dominant” (S1) trajectory, both showing early frontal and motor–cortical atrophy progressing to subcortical structures such as the thalamus and basal ganglia. Their limbic-predominant subtype overlaps anatomically with our “Limbic-Onset with Motor Progression” (S2) trajectory, marked by early hippocampal, amygdalar atrophy; however, our S2 cases showed preserved cognition and higher ALSFRS-R motor scores and lower UMN burden, reflecting a limbic-onset ALS phenotype within the broader ALS-FTD spectrum. In contrast to Shen et al. (2023), our analysis focused on participants without clinical dementia, allowing us to isolate intrinsic motor and limbic heterogeneity specifically among those presenting with ALS, while still capturing a range of extra-motor involvement. Moreover, our DBM-based approach incorporated longitudinal analysis between subtypes and time interval-adjusted mixed-effects modeling when linking SuStaIn-inferred stages to both clinical decline and imaging progression, confirming that inferred trajectories represent reproducible and biologically meaningful disease progression.

Compared with the probabilistic network-based clustering algorithm of Tan et al. (2022) who identified Pure Motor (PM), Fronto-Temporal (FT), and Cingulate-Parietal-Temporal (CPT) clusters using cortical thickness and white matter measures, our SuStaIn-derived subtypes show both overlap and refinement. Our “Motor/CST-Dominant” (S1) subtype aligns closely with Tan’s PM cluster, both reflecting corticospinal, precentral, and brainstem degeneration with concurrent ventricular enlargement. The “Limbic-Onset with Motor Progression” (S2) shares partial limbic-temporal involvement with Tan’s FT subtype but is characterized by relative preservation of frontotemporal cortical integrity and cognition, suggesting a limbic-onset ALS phenotype distinct from the more advanced cognitive phenotypes. Our “Striatal-Fronto-Parietal with Motor-Thalamic Rapid Progressor” (S3) shows the greatest convergence with Tan’s FT cluster, sharing extensive fronto-striatal, thalamic, and motor cortical degeneration, relative cerebellar sparing cross-sectionally, and most severe multidomain cognitive impairment, consistent with the most advanced extra-motor profile within the ALS-FTD spectrum. Overall, our results advance these prior frameworks by integrating temporal staging and longitudinal subtype comparison to distinguish dynamic disease trajectories from static structural clusters.

An advantage of SuStaIn over contrastive trajectory inference and probabilistic clustering approaches is that it explicitly models a Stage 0 (“normal-appearing”) group, representing individuals whose input features remain within the normal range of the inferred disease trajectories. This internally defined baseline enables interpretation of subtype- and stage-specific effects relative to a normative group derived directly from the disease progression model, a feature that is not available in models that derive only disease-associated trajectories or clusters without an explicit zero-stage subgroup.

Associations between SuStaIn stage and both imaging and clinical features confirmed the biological validity of the inferred trajectories. Stage correlated most strongly with atrophy in limbic-striatal and thalamic regions (amygdala, putamen, thalamus, ventral diencephalon), indicating that degeneration in these areas best reflects cumulative disease progression rather than simple aging effects associated with time. These stage–feature associations bridge cross-sectional and longitudinal perspectives: while SuStaIn infers progression from population-level heterogeneity, mixed-effects models capture within-person temporal change. Regions whose atrophy remained significantly correlated with stage after adjusting for ΔTime can thus be interpreted as genuine markers of disease advancement, distinguishing pathological staging from linear aging or follow-up duration. Parallel associations with clinical metrics reinforced the biological interpretability of SuStaIn staging. In the Motor/CST-Dominant subtype (S1), higher stages correlated with lower ALSFRS-R, FVC, and tapping values and greater LMN burden, reflecting progressive decline in functional, respiratory, and motor–neuronal integrity. These relationships confirm that SuStaIn stage meaningfully indexes disease advancement along the motor axis. Paradoxical finger tapping associations with stage in S3 likely stem from sample imbalance and sparse representation at advanced stages rather than true inverse trajectories. Together, these findings indicate that SuStaIn stage captures the expected pattern of motor and respiratory deterioration across ALS progression, providing a biologically interpretable bridge between imaging-inferred stage and functional decline.

This study leveraged a fully data-driven SuStaIn approach applied to DBM-derived volumetric features in regions defined by Brettschneider’s neuropathological staging, yielding biologically interpretable subtypes aligned with known ALS mechanisms. Although based on cross-sectional imaging input, longitudinal validation of subtype stability and progression differences demonstrates temporal robustness and clinical relevance. Notably, this is the first longitudinal validation of a DBM-based SuStaIn model in ALS, providing both mechanistic and clinical evidence of its translational potential for patient stratification and trial enrichment.

There are implications from our findings for individualized patient care and therapeutic targeting. Beyond cohort-level stratification, SuStaIn-derived ALS subtypes may provide a framework for individualized disease monitoring and management. Determining a patient’s subtype from baseline MRI could help anticipate patterns of motor progression, extra-motor involvement, and cognitive decline, thereby informing patient-specific monitoring strategies. For example, individuals classified as S0 may demonstrate relatively preserved brain structure with subtle functional or cognitive changes, suggesting a monitoring-focused strategy emphasizing longitudinal clinical and imaging follow-up. Patients in the S1 (“Motor/CST-Dominant”) subtype may follow a predominantly motor trajectory consistent with classical ALS progression, supporting early emphasis on motor function monitoring and interventions addressing motor disability. In contrast, individuals in the S2 (“Limbic-Onset with Motor Progression”) subtype may benefit from earlier surveillance of limbic-associated symptoms, including mood or behavioral changes, alongside standard motor care. Patients classified as S3 (“Striatal-Fronto-Parietal with Motor-Thalamic Rapid Progressor”) exhibit more extensive fronto-subcortical degeneration and poorer survival, suggesting the potential need for closer multidisciplinary follow-up, earlier cognitive screening, and proactive supportive care planning. At the research level, subtype identification could also support clinical trial enrichment, enabling therapies targeting specific pathological mechanisms or neural systems to be tested in more biologically homogeneous patient groups. In this way, SuStaIn-derived subtype and stage assignments could serve as imaging biomarkers to guide patient stratification, monitoring strategies, and alignment between therapeutic mechanisms and patient-specific disease trajectories. Prospective studies will be required to validate the feasibility and clinical utility of this approach.

Limitations include modest sample sizes within certain subgroups (S2, S3), which may reduce power to detect subtle differences. Additionally, the limited longitudinal observations at advanced disease stages may lead to less robust slope estimates in the association analyses. Furthermore, DBM relies on nonlinear registration and provides a global measure of structural change, without distinguishing cortical surface alterations from more specific white matter tract microstructural changes. Although this approach is robust and well suited for multicenter T1-weighted datasets, complementary imaging measures such as cortical thickness or diffusion-based metrics could provide more specific characterization of cortical and white matter pathology. Future studies integrating multimodal imaging together with genetic and molecular biomarkers may, therefore, further refine subtype-specific mechanisms of ALS progression. The relative hypertrophy observed in limbic and basal nuclei within the Normal Appearing group (S0) also warrants further biological investigation to determine whether this reflects compensatory neural reorganization or methodological factors. Additionally, our analyses were limited to brain MRI measures and thus primarily reflect UMN-related degeneration, without capturing LMN pathology in the spinal cord or peripheral motor pathways. Such early LMN involvement may underlie the S0 subtype, where individuals appear structurally preserved at baseline but later develop motor-related atrophy, highlighting an important avenue for future research. Although this study leveraged a substantial multi-center longitudinal cohort of 198 ALS patients with follow-up assessments at 0, 4, and 8 months, larger and longer-term datasets will be important to confirm the reproducibility of these trajectories and better characterize transitions across SuStaIn stages over time.

Collectively, these strengths, including the multi-cohort design, DBM-based sensitivity, longitudinal SuStaIn validation, ROI selection informed by neuropathological staging and prior neuroimaging evidence, and explicit linkage between imaging-derived stages and clinical progression, advance ALS subtyping toward a dynamic precision-medicine framework for patient stratification, individualized disease monitoring, and mechanism-informed therapeutic development.

## Supplementary Material

Supplementary Material

## Data Availability

Imaging and clinical data can be requested from the CALSNIC consortium (https://calsnic.org/). The scripts for MRI data preprocessing and DBM calculations are available at https://github.com/VANDAlab/Preprocessing_Pipeline. The python SuStaIn code is available at https://github.com/ucl-pond/pySuStaIn. The following open access tools were employed: MINC (https://github.com/BIC-MNI/minc-tools) and ANTs (http://stnava.github.io/ANTs/).

## Appendix 1 Membership of the Canadian ALS Neuroimaging Consortium (CALSNIC).

**Table:**

Name	Title	Affiliation
Dr. Sanjay Kalra	Principal investigator	University of Alberta, Edmonton, AB, Canada
Dr. Christopher Hanstock	Principal investigator	University of Alberta, Edmonton, AB, Canada
Dr. Alan Wilman	Principal investigator	University of Alberta, Edmonton, AB, Canada
Dr. Dean Eurich	Principal investigator	University of Alberta, Edmonton, AB, Canada
Dr. Christian Beaulieu	Principal investigator	University of Alberta, Edmonton, AB, Canada
Dr. Yee Hong Yang	Principal investigator	University of Alberta, Edmonton, AB, Canada
Dr. Lawrence Korngut	Principal investigator	University of Calgary, Calgary, AB, Canada
Dr. Richard Frayne	Principal investigator	University of Calgary, Calgary, AB, Canada
Dr. Hannah Briemberg	Principal investigator	University of British Columbia, Vancouver, BC, Canada
Dr. Lorne Zinman	Principal investigator	University of Toronto, Toronto, ON, Canada
Dr. Agessandro Abrahao	Principal investigator	University of Toronto, Toronto, ON, Canada
Dr. Simon Graham	Principal investigator	University of Toronto, Toronto, ON, Canada
Dr. Angela Genge	Principal investigator	McGill University, Montreal, QC, Canada
Dr. Annie Dionne	Principal investigator	Université Laval, Quebec City, QC, Canada
Dr. Nicolas Dupré	Principal investigator	Université Laval, Quebec City, QC, Canada
Dr. Christen Shoesmith	Principal investigator	Western University, London, ON, Canada
Dr. Michael Benatar	Principal investigator	University of Miami, Miami, FL, United States
Dr. Robert Welsh	Principal investigator	University of Utah, Salt Lake City, UT, United States
